# A Study of Global Quality Scale and Reliability Scores for Chest Pain: An Instagram-Post Analysis

**DOI:** 10.7759/cureus.45629

**Published:** 2023-09-20

**Authors:** Jerusha Deepthi Gudapati, Ancy Jenil Franco, Sweta Tamang, Amir Mikhael, Mohammed Abdul Hadi, Vivek Roy, Majed AlGhoul

**Affiliations:** 1 Internal Medicine, NRI Academy of Medical Sciences, Guntur, IND; 2 Internal Medicine, Sri Muthukumaran Medical College, Chennai, Tamil Nadu, IND; 3 Internal Medicine, Stupa Community Hospital, Kathmandu, NPL; 4 Internal Medicine, College of Medicine, Zagazig University, Zagazig, EGY; 5 Internal Medicine, St Martinus University Faculty of Medicine, Willemstad, CUW; 6 Internal Medicine, Mediclinic Al Noor Hospital, Abu Dhabi, ARE

**Keywords:** social media, reliability score, global quality score, instagram, chest pain

## Abstract

Introduction: Utilized in the healthcare sector, social media offers numerous benefits. However, its drawbacks encompass the variable quality of unregulated and unsupervised content. Thus, we aimed to evaluate the information in content related to chest pain found across Instagram and analyze the quality and reliability of chest pain-related content via Instagram posts.

Methodology: Instagram posts with content related to chest pain were analyzed with the help of a structured questionnaire that included the Global Quality Scale (GQS) and DISCERN score (DS). To collect Instagram posts, several distinct hashtags were employed: #chestpain, #chestpains, #angina, #anginatreatment, #heartattack, #heartattacksurvivor, #heartattackprevention.

Results: A total of 262 posts were included, of which 29.7% of the total posts (n=78) contained information that describes the etiology of the disease. 27.8% of the total posts (n = 73) enclosed promotional content. Posts were found to be uploaded by doctors (18.7%), hospitals (15.6%), patients (17.9%), dieticians (11.1%), healthcare organizations (9.2%), and others (27.5%). Both Global Quality and DISCERN scores were statistically significant with a p-value of 0.001.

Conclusions: The findings of this study revealed that most of the Instagram content on chest pain posted via physicians were highly reliable and had a better global quality score. Information regarding various causes, symptoms and preventive measures on Instagram can be considered as an acceptable source for patients to surf on. A major limitation is that only English content was analyzed. In the future, the use of higher quality posts produced by healthcare professionals could potentially contribute to enhancing patient education via Instagram.

## Introduction

Chest pain with a cardiac cause is called angina pectoris. It comes from the Latin verb "anger," which means to strangle. Annually, a significant number of invasive coronary angiograms are conducted, accompanied by numerous new diagnoses of angina. The use of healthcare resources is significant, with over 110,000 inpatient sessions per year resulting in significant morbidity [1]. The known risk factors for angina are as follows: high serum cholesterol, high-density lipoprotein (HDL) and triglyceride levels, smoking, hypertension, diabetes, family history of acute myocardial infarction, increased thickness of the carotid-artery intima-media, high waist circumference and waist-to-hip ratio, obesity, race and ethnicity [2].

A substantial portion of the American population actively seeks health-related information on social media platforms like Instagram, Facebook, and YouTube, which can impact their decision to seek medical care. However, online information is not always correct, and it does not always originate from reliable sources [3]. As we see the expansion of social media use among the general public around the world, healthcare professionals have also embraced it. It is also reflected in a significant increase in research on social media use in health and medicine, functions of social media in connecting patients and health care professionals, as well as the use of social media for communication among health care professionals [4]. Therefore, our goal was to analyze the content and sources of the top and recent posts related to Angina on Instagram, the most popular photo-sharing site with 2 billion monthly active users.

This study aims to assess the reliability and quality of the information on angina on Instagram using the DISCERN score (DS) and the Global Quality Scale (GQS) score, respectively.

Aims and objectives

The main objective is to evaluate Instagram posts thoroughly, paying close attention to their characteristics, the information they contain, and the general accuracy and dependability of material on the subject of “chest pain”. The GQS score was used as a valid criterion to assess the quality along with the DS to evaluate the accuracy and dependability of the information shared.

## Materials and methods

This study uses a cross-sectional observational methodology, and data was collected on Instagram over the course of two days on August 4 and 5, 2023. Each author was assigned a specific hashtag: #chestpain, #chestpains, #angina, #anginatreatment, #heartattack, #heartattacksurvivor and #heartattackprevention and the top 100 posts were examined.

Posts with English language and titles and posts with informative or instructive content pertaining to patients or public education were included in this study. Posts in any other languages, and posts intended for medical students' education were excluded.

Thus, the following factors were taken into consideration when analyzing posts: the type of post, the number of likes and comments, the amount of time since the post was made, the number of followers that Instagram account has, the type of uploader, and the post's content (details about the disease, its symptoms, prevalence, causes, diagnosis, prevention, treatment, mortality, rehabilitation, and support groups).

The quality and reliability of the Instagram posts were assessed using GQS score and modified DS, respectively [3, 5]. The GQS has five points: 1) Poor quality, poor flow of the site, most information missing, not at all useful for patients; 2) Generally poor quality and poor flow, some information listed but many important topics missing, of very limited use to patients; 3) Moderate quality, suboptimal flow, some important information is adequately discussed but others poorly discussed, somewhat useful for patients; 4) Good quality and generally good flow, most of the relevant information is listed, but some topics not covered, useful for patients; and 5) Excellent quality and excellent flow, very useful for patients) [3]. Modified DS has five questions; a "yes" answer is scored as "1" and "no" answer is scored as "0". The total "yes" answers are calculated to reach a reliability score. These five questions are: 1) Are the aims clear and achieved? ; 2) Are reliable sources of information used?; 3) Is the information presented balanced and unbiased?; 4) Are additional sources of information listed for patient reference?; and 5) Does it refer to areas of uncertainty? [5].

For further analysis, data was exported to a Microsoft Excel file and statistical analysis was done using SPSS version 21. Kruskal-Wallis test was employed to identify any statistical significance between the DS and GQS score.

## Results

The study involved seven authors who individually reviewed 100 posts each, spread across two days (August 4 and 5, 2023), resulting in a cumulative total of 700 posts considered for analysis. After applying stringent inclusion and exclusion criteria, a subset of 262 posts was chosen for further scrutiny.

Presented in Table 1 is a comprehensive breakdown of the posts included in the study, categorized by hashtags. Notably, the hashtags #chestpain and #heartattackprevention yielded the most pertinent outcomes related to the study's focus. Conversely, the hashtags #angina and #anginatreatment provided the least relevant content.

**Table 1:** Total posts evaluated and included in the study

S.no	Hashtag	Posts analyzed	Posts included
1	#angina	100	13
2	#anginatreatment	100	22
3	#chestpain	100	63
4	#chestpains	100	34
5	#heartattack	100	30
6	#heartattackprevention	100	61
7	#heartattacksurvivor	100	39
	Total	700	262

Table 2 takes a closer look at the underlying characteristics of the posts. Impressively, a significant percentage (69.08%) of the posts incorporated visually engaging elements such as digitally created images or videos. The most common form of content was images, accounting for 64.5% of the submissions, while videos or reels constituted the remaining 35.5%. Furthermore, a noteworthy trend emerged as contributions from sources other than doctors, hospitals, patients, dietitians, and healthcare groups accounted for the dominant share of content at 27.5%.

**Table 2:** Characteristics of posts (n=262)

Characteristic	Number (%)
Type of posts	
Image	169 (64.5%)
Video or reel	93 (35.5%)
The total number of audiences reached	
Number of likes	122,580
Number of comments	2743
Number of followers	10,586,686
Is it a digitally created image or video?	181 (69.08%)
Type of uploader	
Doctor	49 (18.7%)
Hospital	41 (15.6%)
Patient	47 (17.9%)
Dietician	29 (11.1%)
Healthcare organizations and health-related websites	24 (9.2%)
Others	72 (27.5%)

The content conveyed through the posts is elucidated in Table 3. Predominantly, the topics of causation (29.77%), prevention (28.24%), and symptom descriptions (27.1%) were the most prevalent. Notably, a significant portion of the posts (27.86%) constituted promotional material disseminated by pharmaceutical companies and medical practitioners.

**Table 3:** Types of information shared

Criteria	Number (Percentage)
Disease description	54 (20.61%)
Symptoms description	71 (27.1%)
Prevalence or incidence-related information	28 (10.69%)
Cause or etiology information	78 (29.77%)
Diagnosis-related information	48 (18.32%)
Preventive measures information	74 (28.24%)
Treatment-related information	69 (26.34%)
Mortality-related information	13 (4.96%)
Rehabilitation information	24 (9.16%)
Support groups information	17 (6.49%)
Information on people or patients expressing their own experiences	50 (19.08%)
Information about patients sharing their experiences with family members	6 (2.29%)
The post includes promotional content by a pharmaceutical manufacturer or from doctors	73 (27.86%)

Table 4 outlines the evaluation of the posts' quality and reliability based on the uploader. The median GQS score is registered at 4 for posts attributed to doctors. Posts emanating from hospitals, dieticians, and other contributors shared a median GQS score of 3, whereas those by patients and healthcare organizations demonstrated a median GQS score of 1. A highly significant p-value of 0.001 demonstrates the considerable difference in GQS scores between doctors and healthcare organizations and patients.

**Table 4:** Assessment of quality and reliability of posts based on the uploader Values are written as median (Q1, Q3), where Q is Quartile. P-value < 0.05 is significant. GQS: Global Quality Scale

Type of uploader	GQS score median (Q1, Q3)	Reliability score (DISCERN) median (Q1, Q3)
Doctor	4 (3, 4)	3 (3, 4)
Hospital	3 (2, 4)	3 (1, 4)
Patient	1 (1, 2)	1 (1, 1)
Dietician	3 (2, 4)	3 (2, 4)
Healthcare organizations and health websites	1 (1, 2)	1 (1, 1.75)
Others	3 (2, 3)	2 (1, 3)
P-value (method: Kruskal-Wallis test)	<0.001	<0.001

Furthermore, the median reliability score (DISCERN) for posts generated by doctors, hospitals, and dieticians registered at 3, whereas posts from other sources averaged at 2, and those from patients and healthcare organizations at 1. This difference in reliability was also statistically significant (p-value = 0.001), indicating that content produced by doctors, hospitals, and dieticians tends to be more reliable than that of patients and healthcare organizations.

The Kruskal-Wallis test was employed to scrutinize the data, with the resulting p-values indicating an extremely significant relationship (p < 0.001). This signifies that posts authored by doctors possess a notably higher GQS score, and posts from doctors, hospitals, and dieticians exhibit superior reliability when compared to those from patients and healthcare organizations.

## Discussion

The importance of social media in spreading public health information and illness preventive recommendations has been highlighted in recent years by medical journals [6]. Since 2013, an array of new uses of social media have emerged, that they primarily benefit people who are ill or who need health information, while the utilization of social media by government entities may benefit everyone in the community, particularly during disease outbreaks. Recent research has started to look into how people outside of patients, the general public, and health professionals utilize social media for health-related goals. Studies have focused on how governmental organizations, such as the US federal, state, and municipal health departments, as well as non-governmental health groups, use social media [7].

The 700 posts included in this study attracted a lot of attention, as evidenced by the 122,580 likes, 2,743 comments, and 10,586,686 followers of these posts, which shows that they were seen by a sizable audience. The findings of a study by Neely, Eldredge, and Sanders titled "Health Information Seeking Behaviors on Social Media during the COVID-19 Pandemic among American Social Networking Site Users" revealed a significant reliance on social media during the COVID-19 pandemic. More than 75% of respondents (762/1,003, 76%) reported that they have relied on social media at least "a little," and 59.2% (594/1,003) of respondents indicated that they read information about COVID-19 once a week [6]. In order to investigate the quality and reliability of Instagram posts on "chest pain," a two-day study was carried out in which each author examined 100 posts, for a total of 700 posts. 262 postings were considered for this study after they met the inclusion and exclusion requirements.

In our analysis, doctors posted 18.7% of the posts, hospitals submitted 15.6%, healthcare organizations and websites uploaded 9.2%, and other groups uploaded 27.5%. This is consistent with a study by Campbell et al. titled "Social Media Use by Physicians: A Qualitative Study of the New Frontier of Medicine," which finds that "uncertainty remains regarding roles and responsibilities of physicians providing medical content within social media forums and few providers appeared to be using the platform to its full potential" [8].

Our study's examination of health information in relation to disease descriptions, symptoms, incidence etiology, and diagnoses was limited, which highlights the significance of providing effective healthcare communication by medical experts as, in a USA poll conducted by Mediabistro, more than 50% of participants claimed to have changed their disease treatment or preventative practices as a result of readings they found online. Additionally, more than 40% of respondents claimed they were likely to change their minds after looking for information online [9].

In our survey, 28.24% of the posts were about prevention, which is comparable to the study on obesity conducted by Aiman et al., where 25% of posts were about preventing obesity, indicating that fewer videos communicate preventative messages [10]. In order to spread concise messages to specific audiences, public health authorities may utilize social media platforms as an effective method to raise public health awareness. However, further studies are required to confirm how social media platforms may be utilized to increase health literacy and the adoption of healthy habits in a cross-cultural setting [11].

According to our analysis, the information about the online support groups was very less, of about 6.49%, which indicates the need for increased support groups as observed in a study done by Plinsinga et al., where a total of 415 people with osteoarthritis completed the survey for the preference of support groups, and 307/415 (73.9%) of the participants have shown interest in support groups [12].

Users of social media may monitor, exchange, and watch each other's health statuses and activities, which have also been observed in our study, that information about people sharing their own experiences was 19.08%. Patients may find that talking and writing about their experiences can help them cope with it and reach their health objectives. Other users may profit from such sharing as it helps them to identify peers with comparable experiences seek advice on changing their lifestyles and explore options for therapy [7].

In line with the findings of Ben O'Mara's study, a significant portion of our study (roughly 69.08%) shows that health information has been disseminated via digitally produced images or videos. This finding supports the notion that digital technology can and does play a significant role in health promotion and in mediating the social determinants of health [13].

In our investigation, 27.86% of the posts were advertisements for pharmaceutical businesses or medical professionals. The requirement for digital ways of advertising medical care services in order to grow a business was shown in research by Radu et al. on the adaptation of healthcare marketing to the digital era, which shows that from January 2015 to October 2016, a total of 22 months, they had witnessed 126 new patients who never received any treatment at this facility, were encouraged by the Internet to look for dental care services. This study made clear how crucial a role social networking sites play in advertising [14].

In our study, doctors' contributions had a considerably higher GQS score than posts from healthcare organizations and other groups, which shares a similar observation in a study done by Rudisill et al. on YouTube as a source of information on pediatric scoliosis: a reliability and educational quality analysis, where JAMA scores for videos uploaded by patients were considerably lower (p = 0.004) [15]. On the other hand, videos uploaded by academics or doctors had higher perceived stress scale (PSS) scores (p = 0.003) and showed a tendency toward better GQS score (p = 0.051) also in keeping in line with a related Instagram analysis research by Aiman et al., which revealed doctors had a large percentage of accurate postings [10].

Based on DISCERN rating in our investigation, it was statistically determined that the posts uploaded by doctors, hospitals, and dieticians were more reliable than those by patients. This is similar to the result of a study by Kunze et al. titled "Quality of Online Video Resources Concerning Patient Education for the Meniscus", which is a YouTube-based quality control study that found that there was significant difference between-group effects for the JAMA score based on the source of the video upload (P .001), with the mean JAMA scores for videos posted by doctors being the highest [16].

Limitations

Posts that weren't in Hindi or English were disqualified as a part of the exclusion criteria. Given that the average Instagram user would often just read through the first 100 posts about chest pain since it can get overwhelming, each person was only given a total of 100 posts (50 each day). Additionally, there may be interobserver bias in the calculations of the GQS score and reliability score.

## Conclusions

In conclusion, our study demonstrates that Instagram posts produced by medical experts and the healthcare sector are more reliable and accurate than those from other sources. Finally, it is critical that verified information be communicated on social media platforms by responsible individuals, such as medical experts and healthcare organizations. This information should not only be easily understood but also have a high global quality and reliability score. By following these guidelines, we may significantly improve the general public's access to reliable information. This access to reputable information is essential for viewers to make informed decisions. Nonetheless, it is critical to underline that individuals should always see their doctors for diagnoses and treatment recommendations. This concentrated effort has the potential to significantly contribute to a better-educated and health-conscious society.
